# Morphological Measurement of Living Cells in Methanol with Digital Holographic Microscopy

**DOI:** 10.1155/2013/715843

**Published:** 2013-01-27

**Authors:** Yunxin Wang, Yishu Yang, Dayong Wang, Liting Ouyang, Yizhuo Zhang, Jie Zhao, Xinlong Wang

**Affiliations:** ^1^College of Applied Sciences, Beijing University of Technology, Beijing 100124, China; ^2^Institute of Information Photonics Technology, Beijing University of Technology, Beijing 100124, China; ^3^College of Life Science & Biotechnology, Beijing University of Technology, Beijing 100124, China; ^4^Beijing Aeronautical Manufacturing Technology Research Institute, AVIC, Beijing 100124, China; ^5^The Pilot College, Beijing University of Technology, Beijing 101101, China

## Abstract

Cell morphology is the research foundation in many applications related to the estimation of cell status, drug response, and toxicity screening. In biomedical field, the quantitative phase detection is an inevitable trend for living cells. In this paper, the morphological change of HeLa cells treated with methanol of different concentrations is detected using digital holographic microscopy. The compact image-plane digital holographic system is designed based on fiber elements. The quantitative phase image of living cells is obtained in combination with numerical analysis. The statistical analysis shows that the area and average optical thickness of HeLa cells treated with 12.5% or 25% methanol reduce significantly, which indicates that the methanol with lower concentration could cause cellular shrinkage. The area of HeLa cells treated with 50% methanol is similar to that of normal cells (*P* > 0.05), which reveals the fixative effect of methanol with higher concentration. The maximum optical thickness of the cells treated with 12.5%, 25%, and 50% methanol is greater than that of untreated cells, which implies the pyknosis of HeLa cells under the effect of methanol. All of the results demonstrate that digital holographic microscopy has supplied a noninvasive imaging alternative to measure the morphological change of label-free living cells.

## 1. Introduction

Cell morphology closely related to its various functions and activities is the research foundation of the modern biomedical discipline and life science. Under the normal cell culture, the size of the living cell changes apparently due to cell proliferation or cell death, and the survival status of the cells can also be estimated by the cell morphology to a great extent. Apoptosis, as a process of the programmed cell death, plays an important role in the development and homeostasis, and the morphological change is a typical feature for distinguishing the apoptosis and necrosis [1, 2]. The cell morphology can also reveal how the living cells have been influenced by the different environmental factors or different medical treatments such as anticancer drugs [3, 4]. Besides, in some diseases such as diabetes mellitus, iron deficiency anemia, and thalassemia, the cell morphology is significantly changed [5, 6]. Comparing with the simple observation, the quantitative phase detection for the morphological change of the label-free living cells has become an urgent demand for the biomedical research. 

Optical microscopy is a major and powerful facility for the biological and medical study for several centuries. Since biological cells are nearly a kind of transparent objects called phase objects, the conventional intensity-based light microscopy imaging method hardly provides the adequate contrast between the cells and the environment. Fluorescence microscopy needs the exogenous label contrast agents such as rhodamine, acridine orange, green fluorescent protein (GFP) to solve the contrast problem, which may make the living cells phototoxic and cytotoxic, and influence the cellular behavior unfortunately [7]. For these issues, many optical phase-imaging methods have been developed to achieve the label-free visual observation of living cells. The phase-contrast imaging techniques such as Zernike phase contrast microscope or differential interference contrast (DIC) microscope apparently increase the contrast of phase or semiphase objects; however, they are inherently qualitative approaches and cannot give the quantitative information of the subcellular structure. Therefore, several techniques have been developed to obtain the full-field and quantitative phase-contrast imaging, such as Fourier phase microscopy (FPM), Hilbert phase microscopy (HPM), diffraction phase microscopy (DPM), and digital holographic microscopy (DHM) [8–13]. Comparing with other imaging methods, DHM has attracted more attention of researches for its particular advantages. DHM can retrieve the quantitative amplitude and phase information of the object wavefront from a single digital hologram, which makes the real-time detection possible. Since the numerical focusing can be implemented by the wave propagation theory, DHM does not demand to strictly record the hologram in the focused image plane of the object, and the digital autofocusing algorithm can help to search the best in-focused image. Furthermore, DHM does not need any complex scanning configuration and possesses a simple setup accordingly.

The noninvasive cell imaging based on DHM has attracted more attention in the biomedical field. Rappaz et al. measured the physiological parameters of the neurons and the testate amoebae by using premagnification digital holography [12, 13]. Kemper et al. studied the invasion mechanism of the living pancreas carcinoma cells and the interaction mechanism of the anticancer drug through the dynamic detection of living cells based on DHM system [14]. Kim et al. achieved the quantitative imaging of ovarian cancer cells through the angular spectrum method. Besides, they also quantitatively studied the wrinkling of a silicone rubber film by motile fibroblasts based on digital holography [15, 16]. Jeong et al. utilized the digital holographic optical coherence imaging to track the effect of cytoskeletal anticancer drugs on tissue inside its natural 3-dimension (3D) environment using time-course measurement of motility within tumor tissue [17]. Pavillon et al. applied the digital holographic microscopy to the early and label-free detection of cell death of the mouse cortical neurons [18]. However, it is also essential to develop the effective setup of DHM and expand its new applications.

The active ingredients of many drugs that are hardly soluble in water can only be dissolved in the organic solvents with high polarity [19, 20]. Methanol, as a kind of organic solvents, is often applied to the *in vitro* pharmacodynamic screening. Nevertheless, the methanol solution with high concentration has toxic effects on living cells; thus the methanol should be diluted to a low concentration when it is used for cell culture. In addition, the fixation of tissues and cells is a key process of immunohistochemistry. Methanol is a frequently used fixative with good penetration [21, 22]. It can remove the lipids leading to cellular dehydration, meanwhile, the proteins instantaneously precipitated on the cytoskeleton. The fixative effects of methanol terminate or reduce the response of exogenous or endogenous enzymes to prevent autolysis of the cells in order to maintain the inherent shape and structure of the tissue cells, more importantly, to preserve antigenicity, and to prevent the loss or diffusion of antigen. In this paper, HeLa cells (human cervix carcinoma cell) are used as the tested sample. The cell morphological change treated with methanol solutions of different concentration is studied by the DHM imaging system. In combination with the numerical analysis and image processing, the surface area and optical thickness of cells are calculated, and the results show that the methanol solutions with different concentrations have diverse effects on the morphology of living HeLa cells.

## 2. Materials and Methods

### 2.1. Principle of Digital Holographic Microscopy

**Figure 1:**
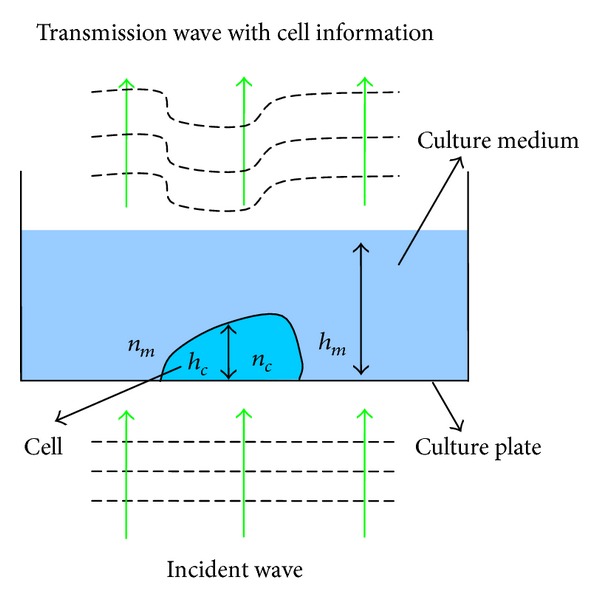
Schematic diagram of the phase imaging.

Digital holographic microscopy, as a quantitative phase-contrast imaging method, is essentially a kind of optical interferometry to detect the phase delay related to the light passing through the tested object. When passing through a relatively transparent sample, the intensity of the light changes very little, while the light through the sample speeds up or slows down and brings a corresponding phase change as indicated in Figure 1. The phase delay or advance depends on the relation of the refraction index between the sample and surrounding environment. Since the phase information is proportional to the optical path length called optical thickness, a depth profile of the tested sample can be calculated. Therefore, digital holography is particularly suitable to measure the phase object such as the living cells and microoptical elements. 

In general, the coherent light source is divided into two arms, one arm goes through the tested object as the object beam, and the other one is used as the reference beam. The interference pattern of the object and reference beams is recorded by a high-resolution CCD detector to obtain the digital hologram represented by

(1) $$\begin{array}{c} I={\left|R\right|}^{2}+{\left|O\right|}^{2}+R^{*}O+RO^{*}, \end{array}$$

where *O* and *R* are the complex amplitude distributions for object and reference waves in the hologram plane, and * denotes the complex conjugate operator.

As shown in (1), |*R*|^2^ and |*O*|^2^ is the dc term, R*O is the real term, and RO* is the virtual term. In the off-axis DHM configuration, the three terms are well separated, and the real term can be extracted by filtering in the frequency domain [23]. Then the real term R*O can be propagated to the image plane using the diffraction theory. Various algorithms have been developed for the numerical reconstruction including Fresnel transform, convolution, and angular spectrum, all of which can be achieved with the help of fast Fourier transform (FFT), and we have analyzed the overlapping quality, accuracy, pixel resolution, computation window, and the speed of these methods [24]. The FFT-based angular spectrum method is used in this paper, which is superior to the FFT-based convolution method in the accuracy and speed. Different from the conventional optical holography, image processing technology can be combined to acquire the quantitative amplitude and phase distribution. In order to obtain the phase distribution from the digital hologram, it is essential to propagate the optical wave to different reconstructed distances to ensure the in-focus plane. The phase aberration induced by the tilted reference wave, microscopic objective (MO), and other optical elements can be corrected. Besides, the phase values are limited in the range of (−*π*, +*π*) due to the principle of the arctan function, so the phase image will contain 2*π* discontinuities when the optical depth of test sample is greater than the wavelength *λ*. The least-squares phase-unwrapping algorithm is a good alternative to acquire the phase information [25]. After deducing the object complex amplitude distribution, we can obtain the phase-contrast image. 

Next, the relation of the optical thickness and the physical thickness is discussed. For the adherent living cell is typically immersed in the cell culture solution as shown in Figure 1, the total optical path delay (OPD) of the transmission wave can be expressed as [12]:

(2) OPDt(x,y)=(n−c(x,y)−nm)hc(x,y)+nmhm=OPDc(x,y)+OPDm,

where ${\overset{-}{n}}_{c}(x,y)$ is the spatially varying integral refractive index, *n*_*m*_ is the refractive index of the culture solution, *h*_*c*_(*x*, *y*) is the spatially varying thickness of the cell, and *h*_*m*_ is the height of the culture solution. The integral refractive index ${\overset{-}{n}}_{c}(x,y)$ is defined as follows:

(3) $$\begin{array}{c} {\overset{-}{n}}_{c}\left(x,y\right)=\frac{1}{h_{c}\left(x,y\right)}\int_{0}^{h_{c}(x,y)}n_{c}\left(x,y,z\right)dz. \end{array}$$

OPD_*m*_ is a reference OPD and can be measured in the place with no cells before calculating OPD_*c*_. Then, OPD_*c*_ can be converted to the cell thickness distribution by

(4) $$\begin{array}{c} h_{c}\left(x,y\right)=\frac{{\text{OPD}}_{c}\left(x,y\right)}{{\overset{-}{n}}_{c}\left(x,y\right)-n_{m}} \end{array}$$

Lue et al. and Jericho et al. have measured the refractive index of HeLa cells using Hilbert phase microscopy based on the microfluidic devices [26, 27], and the results indicated that the refractive index $\bar{n_{c}}$ is 1.385 ± 0.047. However, a change of the cellular refractive index may happen when seeding the Hela cells in different culture medium [13, 28]; meanwhile, as seen from (4), ${\overset{-}{n}}_{c}(x,y)$ is the function of the spatial coordinate, and the intracellular refractive such as the nucleoli and cytoplasm also possesses different refractive index. In view of the refractive index change caused by the intracellular refractive and the addition of different amounts of methanol in our experiment, we only give the optical thickness of Hela cells to describe the morphological feature.

### 2.2. Digital Holographic System

**Figure 2:**
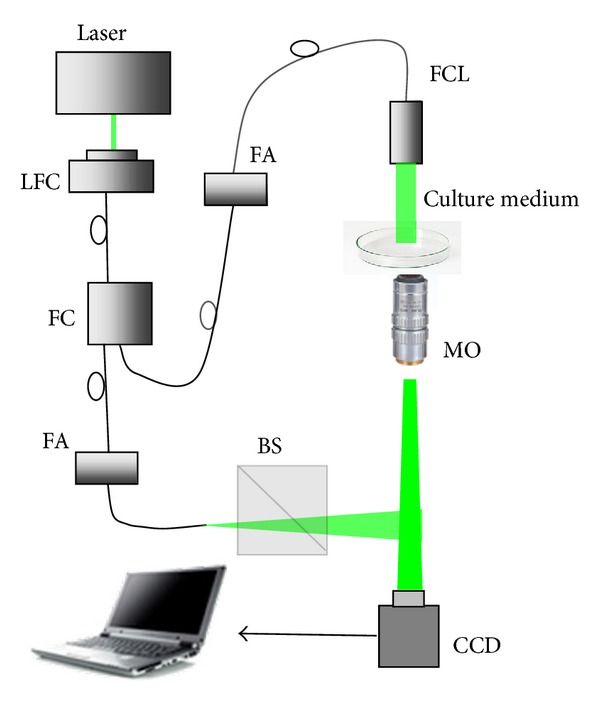
Schematic diagram of setup for the image-plane digital holographic microscopy.

Digital holographic setup can be simplified with the combination of the fiber [29–31]. The image-plane digital holographic microscopy setup is designed as illustrated in Figure 2. The laser source with a wavelength 532 nm is coupled into a fiber by a laser-to-fiber coupler (LFC) and then divided into two arms by a 1 × 2 fiber coupler (FC). A beam collimated by a fiber collimator (FCL) is employed as the object illumination beam, and the other one that is nearly spherical is used as the reference beam. The microscopic objective (20x, NA = 0.4) collects the light transmitted by the sample and produces a magnified real image on the image plane. The CCD camera is placed at the image plane of the object and records the digital hologram. The CCD camera can generate 1280 × 1024 pixel images with 4.65 *μ*m × 4.65 *μ*m sized pixel. The reference light is reflected by a beam splitter (BS), which makes a small angle between the object beam and the reference beam. The two fiber attenuators (FA) in the object and reference arms are applied to adjust the intensity ratio to improve the image quality of digital hologram.

### 2.3. Sample Preparation

HeLa cells (from American Type Culture Collection) were maintained in Dulbecco's modified eagle medium (DMEM) supplemented with 10% (v/v) heat-inactivated fetal bovine serum (FBS), 100 units/mL penicillin, 100 mg/mL streptomycin, and 2 mM L-glutamine. The cells were incubated at 37°C with 5% CO_2_. When the cell confluence reached about 90%, 0.25% trypsin solution was used to digest the cells for five minutes. Then 3.0 × 10^5^ HeLa cells were seeded in 6-well plastic plates. Twenty-four hours after seeding, the cells were about 70% confluence. Methanol solution was serially diluted to 2-, 4-, or 8-fold using DMEM. The cultural supernatant was replaced with the diluted organic solvents or fresh DMEM. Three dilutions were applied, and four duplicates were adopted for each dilution. The concentration of methanol of each dilution was 12.5%, 25.0%, and 50% (v/v), respectively. The wells only with cells and without any organic solvent were used as cell control (CC). Twelve hours later, four samples are used for the morphological analysis of the living cells. 

### 2.4. Digital Processing and Measurements

The hologram recorded by the image-plane digital holographic system is filtered in the frequency domain to remove the dc term and the virtual term. We adjusted CCD to the image plane of the object as much as possible in experiments, whereas it is inevitable that there may be a small distance between CCD and the exact image plane due to the limitation of the tuning component. To compensate the experimental error, the diffraction propagation within a distance ±2 mm is applied to redefine the in-focus plane by angular spectrum algorithm. The phase aberration is corrected using the two-step phase subtraction method. In experiments, a reference hologram without the culture medium is recorded firstly, and then the phase images of the holograms with the culture medium can be acquired. It has been proved that it is sufficient to record a reference hologram prior to the measurement procedure to compensate the phase aberration [32]. Finally, the unwrapped phase image is obtained by the least-squares phase-unwrapping algorithm, and then the quantitative phase information of living cells can be obtained. In order to describe the morphological change of cells, the image segmentation is utilized to extract each cell from the phase image based on Matlab programs. 

The process of the image segmentation is that firstly, the noises are reduced by the Gauss filter and median-filter with 5 × 5 pixels. There are usually a few tens of cells in a phase image, thus a small area including the interested cell is cut from the whole phase image to reduce the computational complexity and improve the accuracy of segmentation. Secondly, the image is enhanced by Sobel operator, and an adaptive threshold algorithm is adopted to transfer the phase image to a binary image. Considering the living cell is generally bigger than the discrete noises, so the connection area of a cell is larger than that of noises. According to this idea, the residual discrete noises can be mostly removed by detecting the pixel number of the connection area. Finally, we can label the location of the interested cell in the binary image. On the one hand, the surface area can be easily calculated according to the total pixel number covered by the cell; on the other hand, we can also obtain the optical thickness of the cell. We pay more attention to the maximum optical thickness and average optical thickness of the cell. It is worth noting that the optical thickness of the cell is the difference between the thickness with cells and the reference thickness without cells. To increase the precision of optical thickness, the reference thickness is computed by averaging the optical thickness of several areas without cells. 

## 3. Results and Discussion

**Figure 3:**
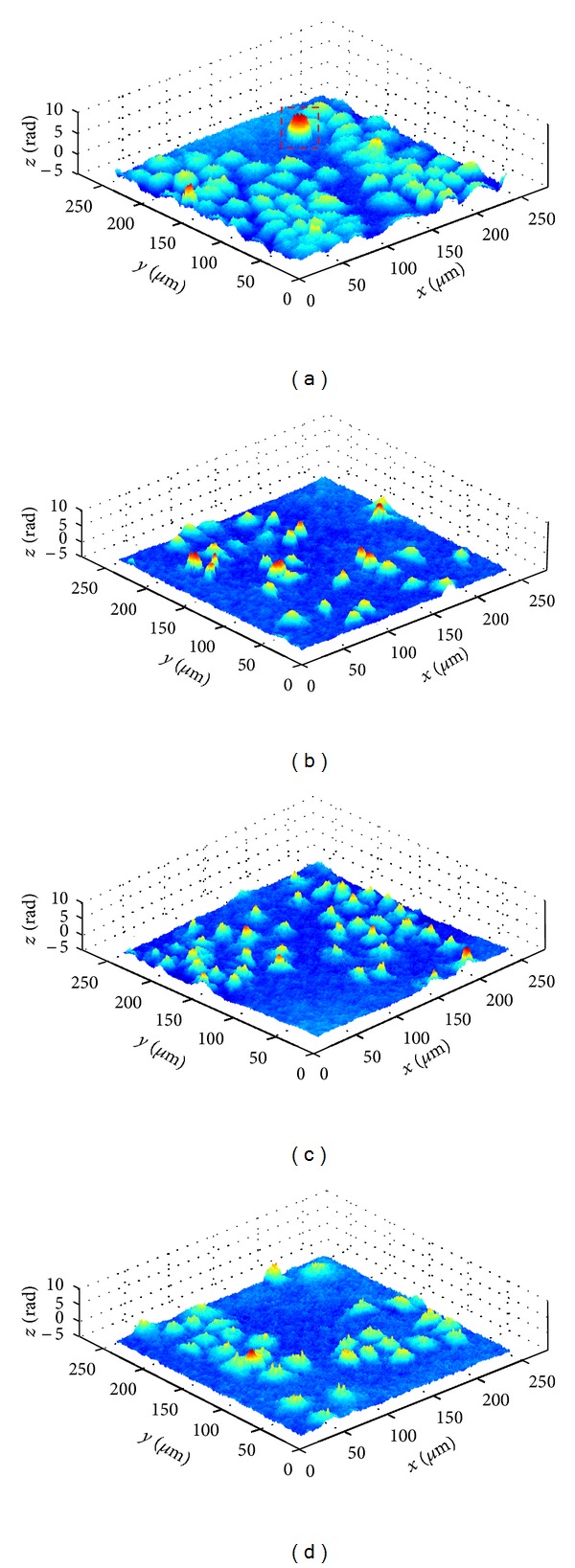
Phase-contrast imaging results of living cells after seeding 12 hours: (a) cell control; (b) treated with 12.5% methanol; (c) treated with 25% methanol; (d) treated with 50% methanol.

In experiments, HeLa cells treated with different concentrations of methanol are imaged in the plastic plate. The morphology of untreated cells is illustrated in Figure 3(a). The cells are arranged regularly with the shape of polygon or diamond, which indicate that cells are mostly growing adherently in a healthy status. Besides, it is worth to note that the spontaneous death or aging of some cells may happen though cultured in a good condition. With the death of aging cells, the cells gradually lose the ability of adherent and become rounded under the surface tension in the solution. As shown in the dashed box of Figure 3(a), it is apparently a typical rounding death cell. Aiming to this kind of cells, not only the surface area changes into a round shape, but also the cell thickness has a visible change that can be supplied by digital holographic technology superiorly. For 18 normal cells in Figure 3(a), their average optical thickness $\bar{\text{OT}}$ is 0.99 rad, and the average maximum optical thickness $\bar{\text{O}{\text{T}}_{\max}}$ is 2.89 rad. Here, the average maximum optical thickness is defined as the average value of the maximum optical thickness of the counted cells. For the rounding cell in the dashed box, its average optical thickness $\bar{\text{OT}}$ becomes higher up to 3.47 rad, and its maximum optical thickness OT_max⁡_ is 6.45 rad. Therefore, the oversize optical thickness may be a characterization of this kind of abnormal cells. 

Methanol solutions with different concentrations have diverse effects on the morphology of living HeLa cells. Since the intermediate metabolite such as formaldehyde and formic acid caused by the excessive methanol will damage the cells to some extent. The cell morphologies treated with 12.5%, 25% and 50% methanol are shown in Figures 3(b), 3(c), and 3(d), respectively. We can see that the cell morphologies treated with methanol have obvious changes, especially the cell morphologies treated with 12.5% and 25% methanol.

**Figure 4:**
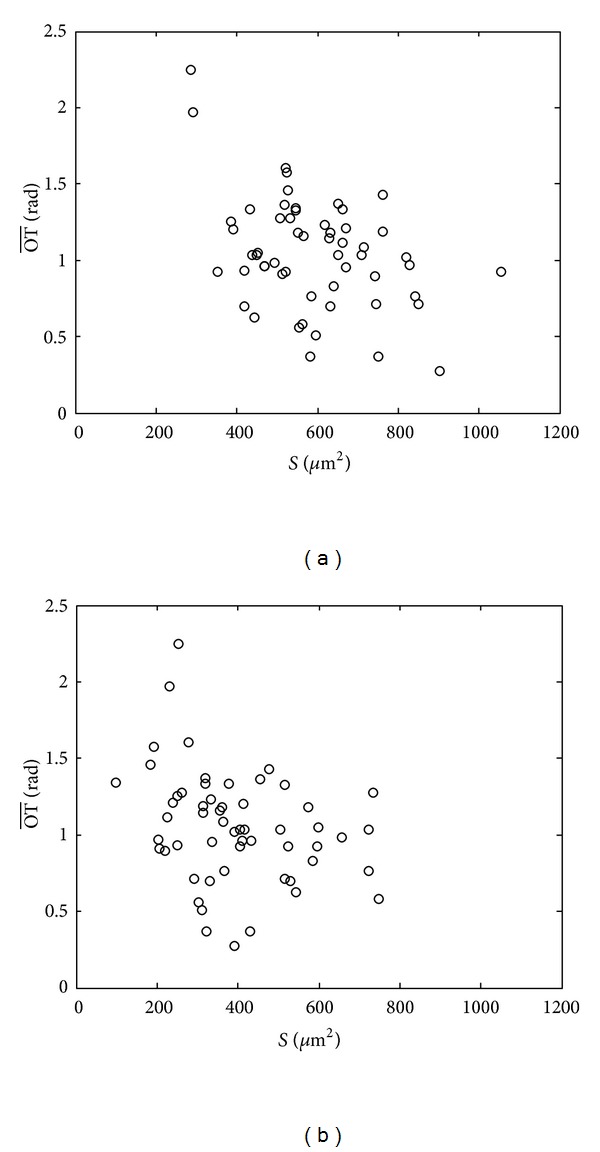
Size distribution of 56 cells: (a) cell control; (b) treated with 12.5% methanol.

The size distribution of 56 cells treated with 0% or 12.5% methanol is depicted in Figure 4 intuitively, where *x* and *y* coordinates represent the surface area (*S*) and the average optical thickness ($\bar{\text{OT}}$) of cells. Comparing Figure 4(a) with 4(b), both the cell area and average optical thickness distribute in a different interval, which imply the shape changes accordingly. 

To describe the shape change quantitatively, the information of 56 cells are extracted from each concentration, and the parameters including area *S*, average optical thickness ($\bar{\text{OT}}$), average maximum optical thickness ($\bar{\text{O}{\text{T}}_{\max}}$), and the respective standard deviations (SD) are listed in Table 1. The statistical significance of experimental data is analyzed using *t* test and (analysis of variance) ANOVA based on Statistical Package for the Social Sciences (SPSS) Release 16.0. ANOVA shows significant differences existing in the above parameters between the four groups (*P* < 0.01). Then differences between the group of cell control and the group treated with methanol were probed. For HeLa cells treated with 12.5% or 25% methanol, the area is reduced significantly compared with that of the normal cells (*P* < 0.01), which indicates that the methanol with lower concentration could cause cellular shrinkage. For HeLa cells treated with 50% methanol, the area is similar with that of the normal cells (*P* > 0.05), which verifies the fixative effect of higher methanol concentration. However, there are still significant differences in both $\bar{\text{OT}}$ and $\bar{\text{O}{\text{T}}_{\max}}$ (*P* < 0.01) that can only be detected by phase image. The morphological feature of cells treated with 25% methanol shows significant differences in three parameters *S*, $\bar{\text{OT}}$, and $\bar{\text{O}{\text{T}}_{\max}}$ (*P* < 0.01). Besides, the average maximum optical thickness of the cells treated with 12.5%, 25%, and 50% methanol is greater than that of untreated cells, which implies the pyknosis of HeLa cells under the effect of methanol. 

## 4. Conclusions

It has been recently an urgent demand to quantitatively detect the morphology for living cells in the biomedical and life science field. In this paper, the morphological change of HeLa cells treated with the methanol solution is measured based on digital holographic microscopy. Methanol, as a kind of organic solvents, is often used to dissolve some drugs with low concentrations and also applied to the fixation of tissues and cells with high concentrations. After recording the hologram using the image-plane digital holographic system, the phase image of living cells is calculated by numerical analysis. With the assistance of the image processing, the surface area and optical thickness of the living cells are computed to describe the cell morphology quantitatively. The ANOVA shows significant differences between the four groups (*P* < 0.01). Compared with the CC group, *S* and $\bar{\text{OT}}$ of HeLa cells treated with 12.5% or 25% methanol are reduced significantly, which verifies that the methanol with lower concentration has the toxic effects and could cause cellular shrinkage. For HeLa cells treated with 50% methanol, *S* is similar with that of the normal cells (*P* > 0.05), which reveals the fixative effect of methanol with higher concentration. Furthermore, $\bar{\text{O}{\text{T}}_{\max}}$ of the cells treated with 12.5%, 25%, and 50% methanol is greater than that of untreated cells, which implies the pyknosis of HeLa cells under the effect of methanol. All of the results demonstrate that digital holographic microscopy is a noninvasive imaging approach for detecting the morphological change of the label-free living cells.

## Figures and Tables

**Table 1:** Data of the cell morphology with different methanol concentration.

Methanol concentration (%)	Parameters		
	*S* (*μ*m^2^)	$\bar{\text{OT}}$ (rad)	$\bar{\text{O}{\text{T}}_{\max}}$ (rad)
0 (CC)	590.3 ± 153.9	1.1 ± 0.4	3.1 ± 0.6
12.5	394.8 ± 152.8**	0.8 ± 0.4**	3.2 ± 0.7
25	488.3 ± 180.6**	0.8 ± 0.3**	4.2 ± 0.6**
50	574.0 ± 211.8	1.3 ± 0.4**	4.1 ± 0.6**

Remark: where **P < 0.01 compared with the group of cell control.
